# Beyond the Plate: Uncovering Inequalities in Fruit and Vegetable Intake across Indonesian Districts

**DOI:** 10.3390/nu15092160

**Published:** 2023-04-30

**Authors:** Ede Surya Darmawan, Dian Kusuma, Vetty Yulianty Permanasari, Vilda Amir, Dwi Hapsari Tjandrarini, Ika Dharmayanti

**Affiliations:** 1 Department of Health Administration and Policy, Faculty of Public Health, Universitas Indonesia, Depok 16424, Indonesia; 2 Department of Health Services Research and Management, School of Health & Psychological Sciences, City University of London, London EC1V 0HB, UK; 3 Center for Health Administration and Policy Studies, Faculty of Public Health, Universitas Indonesia, Depok 16424, Indonesia; 4 Research Center for Public Health and Nutrition, National Research and Innovation Agency, Bogor 16915, Indonesia

**Keywords:** unhealthy diet, dietary risks, inequality, geographic, socioeconomic

## Abstract

Background: Non-communicable diseases (NCDs) are responsible for the deaths of 41 million individuals every year, with 77% of them occurring in low- and middle-income countries. Among the main NCD risk factors, inadequate intake of fruits and vegetables (FV) was one of the leading causes of death in 2019. Our study aims to identify disparities in inadequate FV intake among adults in 514 districts. We utilized the latest Indonesian Basic Health Survey 2018 to conduct geospatial and quantitative analyses. We used the World Health Organization’s definition of inadequate FV intake, which refers to consuming less than five servings of fruit and vegetables daily. We analyzed inadequate FV intake among adults over the age of 18 years, as well as by gender and age group (including young adults 18–24 years, adults 25–59 years, and older adults 60+ years). Our study showed an alarmingly high prevalence of inadequate FV intake among adults, with 96.3% in 2018. The prevalence of inadequate FV intake drastically varied across 514 districts, ranging from 70.1% to 100%. Notable geographic and socioeconomic disparities were observed across the districts studied. Rural districts exhibited a higher prevalence of inadequate FV intake, translating to poorer diets, particularly among females and older adults, when compared to their urban counterparts. Interestingly, districts within more developed regions had poorer FV diets than those in less developed regions. Although districts with lower incomes generally had poorer FV diets, the association was not significant in multivariate analysis. However, districts with lower levels of education demonstrated poorer FV diets, especially among females, adults, and older adults. Despite its limitations, our study provides crucial insights for health policies in Indonesia and other LMICs.

## 1. Background

Non-communicable diseases (NCDs) claim the lives of 41 million people yearly, which constitutes 74% of total global deaths. Out of these fatalities, 77% are in low- and middle-income countries (LMICs) [1]. The two most critical NCDs are cardiovascular diseases (CVDs) and diabetes, causing 17.9 million and 2.0 million deaths, respectively [1]. The latest Global Burden of Disease Study identified CVDs, such as ischemic heart diseases and stroke, and diabetes, as among the top ten leading causes of global deaths and disabilities in 2019 [2]. Additionally, the study noted that dietary risks, including inadequate intake of fruits and vegetables (FV), were among the leading causes of death for both genders in 2019 [3].

Indonesia is currently facing an increasing burden of NCDs, while also dealing with maternal mortality and infectious diseases. According to the latest Basic Health Research (Riskesdas) by the Ministry of Health, the prevalence of NCDs and their risk factors has dramatically increased over the past five years [4]. For instance, the prevalence of diagnosed stroke among individuals aged 15 years and above surged by 56%, from 0.7% to 1.1%, while that of diabetes mellitus increased by 23%, from 6.9% to 8.5%, between 2013 and 2018. Additionally, an overwhelming 95.4% of the population aged 5 years and above had insufficient FV intake, consuming less than the WHO-recommended daily portions [4].

There can be several reasons for deficiencies in the FV consumption. First, fresh FV may not be readily available or accessible in some areas due to inadequate distribution systems or limited availability in local markets [5]. Second, fresh FV can be more expensive than other food options, making it challenging for some individuals to regularly afford them [6]. Third, many people may not be aware of the health benefits, or they may lack knowledge on how to incorporate them into their diets [7]. In addition, some cultural or social factors may discourage the consumption of certain FV or promote the consumption of other foods instead [8]. Fourth, many people may choose convenient, processed foods over fresh FV, which require more preparation and cooking time [9].

Several studies have demonstrated a link between inadequate FV intake and socioeconomic status and geographic location. In a 2019 systematic review that examined 40 studies on individuals aged 10–40 years in high-income countries, it was found that a higher FV intake was associated with better parental socioeconomic status, especially with regard to higher education levels [10]. Furthermore, studies utilizing geospatial analysis in the United States in 2008 revealed that neighborhood socioeconomic status was positively related to FV consumption [11]. An analysis conducted in Brazil in 2017 also discovered that areas with higher incomes and better-quality food stores had higher FV intake [12]. Similarly, a study in 2012, which used data from 232,056 adult participants in 48 LMICs from the 2002–2004 World Health Survey, found that inadequate FV intake was significantly higher among lower socioeconomic groups [13]. Similar findings were also observed in analyses conducted in the Netherlands and Brazil [14,15].

Reducing the inequality in inadequate FV intake is crucial in achieving Sustainable Development Goal Target 3.4.1, which aims to decrease premature mortality from NCDs. However, the current research on disparities in inadequate FV intake among adults has limitations. Firstly, most studies have used individual-level data [13,14,15], which are useful but may not capture locality-level factors in countries such as Indonesia. Secondly, previous research on geographic disparities has focused mainly on high-income countries, leaving limited studies in LMICs [10,11,12,13,14,15,16]. A 2014 study in Ghana compared FV intake across only three regions and found a higher consumption in the Forest zone due to better production [16]. Thus, our study aims to investigate disparities in FV intake among adults across 514 Indonesian districts, considering socioeconomic and geographic factors. Our study intends to fill the gap in the research by analyzing district-level data in Indonesia, a country with significant local decision-making power. Our research will provide valuable insights into addressing inadequate FV intake disparities, particularly in LMICs.

## 2. Methods

### 2.1. Study Design

This study aimed to investigate disparities in inadequate FV intake among adults aged 18 or older in Indonesia using geospatial and multivariate regression analyses. The study analyzed data from the 2018 Indonesian Basic Health Survey (Riskesdas), a nationwide health survey that collected information on FV intake from 514 districts across 34 provinces [4]. The survey aimed to include 300,000 households and utilized a 2-stage sampling method to select 30,000 census blocks in both urban and rural areas, followed by the selection of 10 households in each block based on the education level of the household head [4]. The study utilized both geospatial analysis and multivariate regression to analyze the collected data.

### 2.2. Independent Variables

The study examined four variables: region, urbanicity, income, and education, at the district level, and obtained the data from the World Bank database. The provinces and districts were classified into five regions, including Sumatera, Java and Bali, Kalimantan, Sulawesi, and Papua/Nusa Tenggara/Maluku. Java and Bali are more developed than the eastern regions [17,18,19]. The analysis was conducted for all districts, cities (urban districts), and regencies (rural districts).

In this study, poverty rates were used as a proxy for the income level of districts. The poverty rates for each district were divided into five groups, with the highest poverty rates assigned to the first quintile and the lowest poverty rates assigned to the fifth quintile. The basic needs approach, which is based on the Handbook on Poverty and Inequality published by the World Bank, was used by the Indonesia Statistics Bureau (BPS) to determine poverty levels. This approach defines poverty as the inability, from an economic perspective, to fulfill basic food and non-food needs, which are measured in terms of expenditure. Individuals are considered poor if their average monthly expenditure per capita falls below the poverty line [20]. Similarly, the net enrollment ratios for senior secondary education were grouped into five categories, with the first quintile representing the least educated and the fifth quintile representing the most educated [21,22]. Supplementary Figures S1 and S2 provide the country map and the district-level map by urban/rural, income level, and education level. Supplementary Table S1 shows the urban sample and the levels of education and poverty by urbanicity and region.

### 2.3. Dependent Variables

Our research focused on inadequate FV consumption as the outcome variable among all adults, males, females, young adults (18–24 years), adults (25–59 years), and older adults (60+ years). The FV questionnaire was adapted for use in Indonesia by the Ministry of Health and was based on the World Health Organization (WHO) STEPwise approach to NCD risk factor surveillance (STEPS) questionnaire, which is widely employed in global disease surveillance [23]. First, the respondents were asked about the number of days in a typical week on which they consume fruits (vegetables). Second, they were asked to specify the number of portions consumed in a typical day within the same week. For the analysis, we used the WHO’s definition of inadequate FV intake, which refers to consuming less than five servings of fruit and vegetables daily [24]. We analyzed the prevalence of low intake among males and females separately to understand the differences. Furthermore, we also examined the prevalence of low intake among different age categories, which is crucial for controlling and preventing NCDs and developing effective health system responses [25].

### 2.4. Data Analysis

We utilized geospatial analysis and multivariable regressions to explore the link between inadequate FV intake and geographic/socioeconomic factors. The data were divided into quintiles using ArcMap 10.6 (Esri, Redlands, CA, USA), and we conducted Ordinary Least Square (OLS) regressions in STATA 15.1 (StataCorp LLC, College Station, TX, USA). Our analysis examined the connections between region, urbanicity, income, and education indicators, and each of the six outcome variables. We compared the variations between the most and least developed regions and the poorest and wealthiest/most educated quintiles. A statistical significance level of 5% or lower was deemed significant.

## 3. Results

### 3.1. Provincial-Level Results

**Figure 1:**
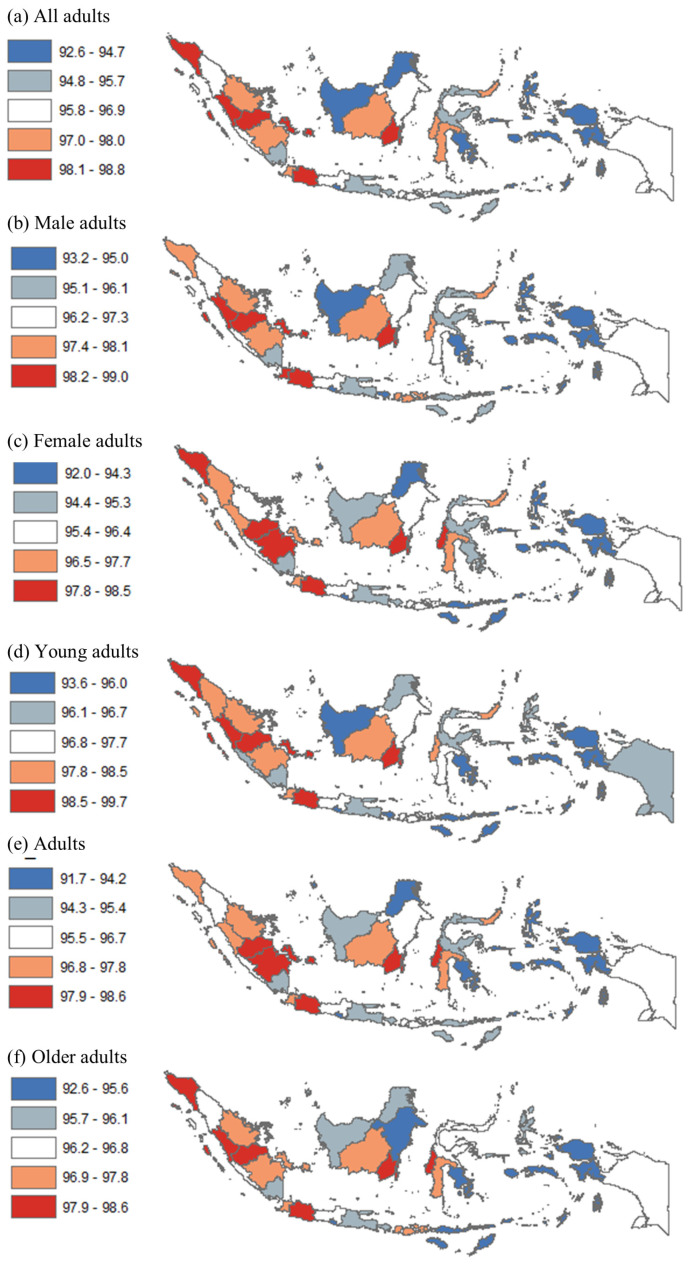
Disparity in inadequate FV intake among adults by province in Indonesia, 2018. FV = fruit and vegetable. Numbers show the prevalence of inadequate FV intake among all adults, males, females, young adults, adults, and older adults. The prevalence was grouped by quintile.

Figure 1 displays the percentage of adults with inadequate FV intake across provinces. Figure 1a–f depict the data for all adults, male adults, female adults, young adults, adults, and older adults. The prevalence of inadequate FV intake varied among different provinces, with percentages ranging from 92.6% to 98.8% among all adults. The ranges were similar by sex (males and females) and age group (young adults, adults, and older adults). Moreover, the provinces with the highest prevalence (quintiles 4–5) of inadequate FV intake among all adults were mostly located in Sumatera, including Aceh, Jambi, Bangka Belitung, West Sumatera, South Sumatera, and Riau, as well as in some provinces in Kalimantan, Sulawesi, and Java. Such pattern was similar across sexes and age groups.

Table 1 displays the prevalence of inadequate FV intake among adults in each province. Sorted by poverty rates, the top box shows that wealthiest and the bottom box shows the poorest ten provinces. Grey-colored cells indicate a higher prevalence than the national average for each column (outcome indicator). Among the top ten wealthiest provinces, five had a higher-than-average prevalence for all six indicators, including South Kalimantan, Central Kalimantan, Banten, Bangka Belitung, and West Sumatera. However, only three of the ten poorest provinces had a higher-than-average prevalence.

### 3.2. District-Level Results

Table 2 presents the descriptive statistics of the districts included in our study, which includes the proportion of adults with inadequate FV intake. Out of the 514 districts, 97 (18.9%) were urban cities, and the remaining 417 (81.1%) were rural regencies. The cities were mainly situated in Java (36.1% of 97) and Sumatera (34.0%), while regencies were less concentrated, with 29.0% (of 417 regencies) in Java, 22.3% in Sumatera, 20.6% in Papua, 16.8% in Sulawesi, and 11.3% in Kalimantan. When analyzed based on income level, it was found that 79% of urban areas belonged to the wealthier group (quintiles 4–5), whereas almost half (47.2%) of the rural areas were part of the poorer group (quintiles 1–2). In terms of education level, 71.1% of cities belonged to the higher education group (quintiles 4–5), while almost half (46.8%) of the regencies were part of the lower education group (quintiles 1–2). Regarding the proportion of adults with inadequate FV intake, it was found that 96.3% of all adults had inadequate FV intake, with males and females having a prevalence of 97.6% and 96.0%, respectively. Moreover, young adults, adults, and older adults had an inadequate FV intake prevalence of 97.0%, 96.1%, and 97.6%, correspondingly. Compared to urban areas, rural areas had a higher prevalence of inadequate FV intake, and this difference was statistically significant in rural areas among females (*p*-value = 0.020) and older adults (*p*-value < 0.001). The prevalence of inadequate FV intake among females and older adults was 95.2% in urban areas, while in rural areas, it was 96.2% and 97.0%, respectively.

**Figure 2:**
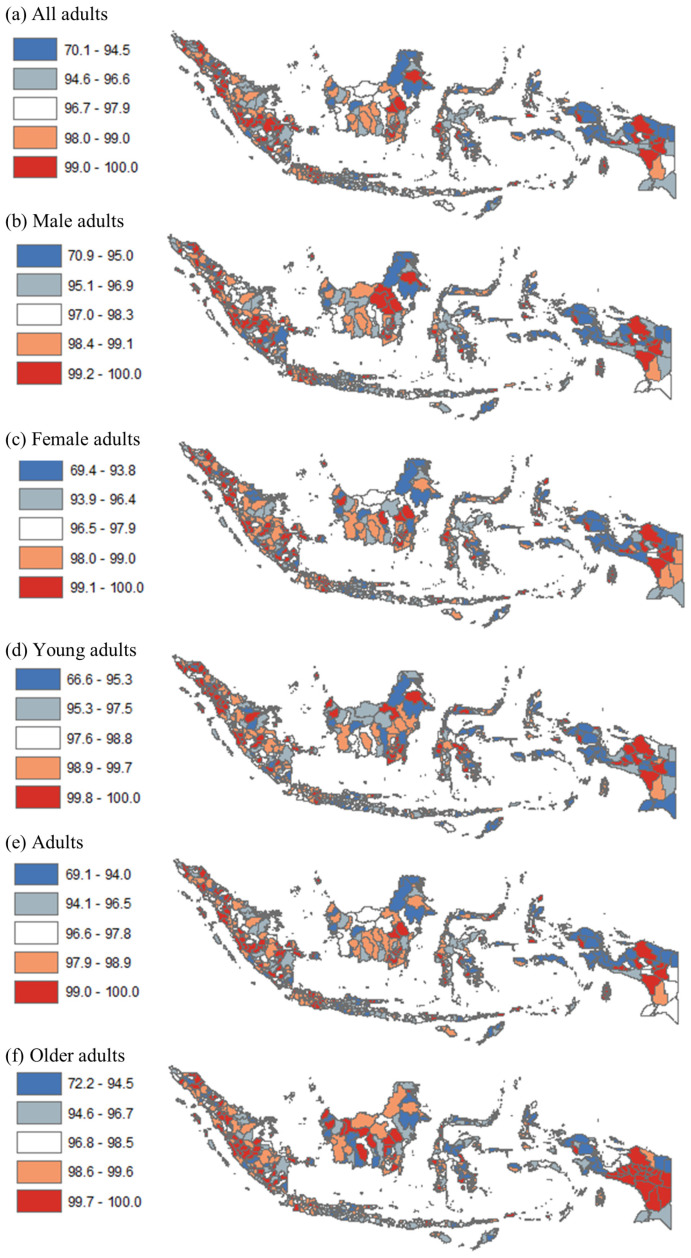
Disparity in inadequate FV intake among adults by district in Indonesia, 2018. FV = fruit and vegetable. Numbers show the prevalence of low FV intake among all adults, males, females, young adults, adults, and older adults. The prevalence was grouped by quintile.

In Figure 2, there is a more detailed breakdown of the prevalence of inadequate FV intake by quintile at the district level. The prevalence of inadequate FV intake varied among different districts, with percentages ranging from 70.1% to 100% among all adults. Figure 2 provides a more detailed view than at the provincial level. For example, despite showing a relatively lower prevalence of inadequate FV intake at the provincial level, many districts in various provinces, such as North Sumatera and Lampung, Central Java and East Java, West and North Kalimantan, and Papua and West Papua, had a high prevalence of inadequate FV intake (quintiles 4–5) among all adults. Similarly, despite showing a relatively higher prevalence at the provincial level, many districts in Riau and Jambi, Central and South Kalimantan, and Banten and West Java had a lower prevalence of inadequate FV intake (quintiles 1–2). This pattern was consistent for the prevalence among males, females, young adults, adults, and older adults.

Supplementary Tables S2 and S3 show the ten districts with the lowest and highest prevalence of inadequate FV intake among adults, respectively, revealing significant socioeconomic disparities. Among all adults, inadequate FV intake ranged from 70.1% in Kolaka Timur (Sulawesi region) to 100% in several rural regencies in the Sumatera, Sulawesi, and Papua regions. Moreover, many districts with the lowest prevalence of inadequate FV intake were rural, while all districts with the highest prevalence were also rural. The average poverty rates among the districts with the lowest prevalence of inadequate FV intake were up to 20%, while the rates among the districts with the highest prevalence were up to 35%.

Table 3 and Table 4 display the associations between geographic (e.g., region) and socioeconomic indicators (e.g., income and education) with inadequate FV intake. Table 3 shows the prevalence of inadequate FV intake by region, income, and education, while Table 4 illustrates the results of multivariable regressions. The prevalence of inadequate FV intake was higher in Sumatera, Java, Sulawesi, and Kalimantan, by 1.69%, 1.86%, 1.18%, and 2.59%, respectively, in contrast to Papua. This trend was consistent across sex and age group. Inadequate FV intake was somewhat greater among districts with higher incomes, although the differences were not statistically significant in the multivariable regression. In comparison to districts with the least education, districts with the most education, especially among females, adults, and older adults, had a significantly lower prevalence of inadequate FV intake, by 1.28%, 1.28%, and 1.25%, respectively.

## 4. Discussion

In 2018, we found an alarmingly high prevalence of inadequate FV intake among Indonesian adults aged 18 and above. At the district level, the prevalence of inadequate FV intake was 96.3%, 97.6%, and 96.0% for all adults, males, and females, respectively. Across age groups, the prevalence was 97.0%, 96.1%, and 97.6% for young adults (18–24 years), adults (25–59 years), and older adults (60 years and above), respectively. These findings are comparable to other LMICs, such as India (95.8%), Pakistan (96.5%), Nepal (99%), Ethiopia (98.5%), and Kenya (94%) [26,27,28,29,30].

Our research revealed that there was a significant disparity in inadequate FV intake among adults in Indonesia based on both geography and socioeconomic status across 514 districts. Rural districts had, on average, a higher prevalence of inadequate FV intake (i.e., poorer FV diet), especially among females and older adults, compared to urban districts. However, note that many districts with the lowest prevalence of inadequate FV intake (i.e., better FV diet) were rural regencies. In high-income countries, FV intake can vary between urban and rural areas due to factors such as access to fresh produce, income, education, and cultural practices. However, there are some general trends that urban areas tend to have greater access to a variety of fruits and vegetables due to the presence of supermarkets, grocery stores, and specialty shops. This can lead to a higher overall consumption of fruits and vegetables in urban areas compared to rural areas [11,31]. In LMICs, FV intake in urban and rural areas can be quite different due to various factors, such as access to markets, availability, affordability, cultural practices, and overall dietary patterns [32]. In many LMICs, urban residents may have better access to markets and a wider variety of fruits and vegetables due to the concentration of shops, supermarkets, and local vendors. However, urban populations can also experience disparities in access to healthy food options, particularly in low-income neighborhoods or informal settlements [27,29]. In rural areas of LMICs, residents might have more opportunities to grow their own fruits and vegetables, leading to increased consumption of fresh produce. However, the diversity of fruits and vegetables available may be limited due to seasonality, geographic constraints, or a lack of market access to a wider variety of fresh produce [16,27,29].

Our study also found that districts in more developed regions (Java, Sumatera, Kalimantan, and Sulawesi) had poorer FV diets than those in less developed regions (Papua, Maluku, and Nusa Tenggara). In LMICs, FV intake can significantly vary between regions and within communities due to various factors. Some less developed regions might have a lower FV intake due to factors such as limited access to markets, lower income levels, and fewer diverse food options. Analysis among 18 countries from the Prospective Urban Rural Epidemiology (PURE) study found the cost of two servings of fruits and three servings of vegetables per day per individual accounted for 52.0% of the household income in low-income countries, 18.1% in lower-middle income countries, 15.9% in upper-middle income countries, and 1.9% in high-income countries [33]. However, local agricultural practices and traditional diets in some LMICs might lead to higher levels of FV consumption. A 2014 study in Ghana found that the FV intake was notably higher in the Forest zone, where the production of fruits and vegetables was more advanced compared to the Savannah and Coastal zones [16].

Our findings showed that districts with lower incomes tended to have poorer FV diets, but the associations were not significant in the multivariate regression. Moreover, we found that districts with less education had poorer FV diets, particularly among females, adults, and older adults. These results align with previous studies. A geospatial analysis from the United States found that poorer socioeconomic status at the neighborhood level was associated with lower FV intake [11]. Another study in Brazil showed that average FV intake was higher in neighborhoods with higher incomes and concentration of food stores, and a better index of access to healthy foods [12]. Similarly, a multilevel study in the Netherlands found significant disparities in FV intake, with odds ratios of not consuming FV at 5.47 among the lowest-educated groups [14].

For policy, inadequate FV intake among adults in Indonesia is a significant public health concern and is associated with high rates of CVDs and diabetes, and their main risk factors, such as obesity and hypertension [18,22,34]. There are several policy implications that can be drawn. First, policymakers should consider incorporating the study’s evidence-based insights into the development of targeted interventions, in order to bridge the gap in FV intake between rural and urban areas, as well as between different socioeconomic groups in Indonesia. This may include enhancing access to diverse and affordable FV options in rural and low-income areas through improved transportation and market infrastructure, as well as supporting local agricultural practices [35,36]. Additionally, public health campaigns should emphasize the importance of FV consumption and aim to increase awareness, particularly among females, older adults, and those with lower levels of education. These campaigns can be tailored to the cultural practices and dietary patterns of specific regions, taking into account the unique challenges faced by communities in both developed and less developed areas [32,33]. Lastly, further research is needed to identify and address barriers to FV intake in specific districts and to evaluate the effectiveness of interventions in improving dietary habits among the Indonesian population.

Our study is the first to investigate disparities in inadequate FV intake among adults across more than 500 districts. However, it has at least three limitations, which may be due to at least two reasons. Firstly, we were unable to examine a sub-group analysis by ethnicity as we lacked relevant data in our dataset [37]. Secondly, the study was conducted using cross-sectional data, and trends over time could not be assessed. Thirdly, there was only a small urban area (i.e., 95 cities of 514 districts), which may limit the comparison. Our definition of urban/rural (cities = urban and regencies = rural) may not capture the most accurate variations in terms of more/less developed regions as well as education/income levels. For instance, some regencies that are adjacent or closer to cities may have a similar development/education/income level to the cities. Nevertheless, our findings are essential for informing health policies in Indonesia and other LMICs.

## 5. Conclusions

Our study in Indonesia showed an alarmingly high prevalence of inadequate FV intake among adults, with 96.3% in 2018. The prevalence of inadequate FV intake drastically varied across the 514 districts, ranging from 70.1% to 100%. Notable geographic and socioeconomic disparities were observed across the districts studied. Rural districts exhibited a higher prevalence of inadequate FV intake, translating to poorer diets, particularly among females and older adults, when compared to their urban counterparts. Interestingly, districts within more developed regions had poorer FV diets than those in less developed regions. Although districts with lower incomes generally had poorer FV diets, the association was not significant in multivariate analysis. However, districts with lower levels of education demonstrated poorer FV diets, especially among females, adults, and older adults. Although our study has limitations, it provides crucial insights for health policies in Indonesia and other LMICs.

## Figures and Tables

**Table 1:** Prevalence of inadequate FV intake among adults by province in Indonesia, 2018.

		Low FV Intake Prevalence (%)					
	Poverty Rates (%)	All	Males	Females	Young Adults	Adults	Older Adults
	(1)	(2)	(3)	(4)	(5)	(6)	(7)
Bali	4.5	94.8	95.0	94.5	95.6	94.4	95.6
South Kalimantan	4.8	98.8	99.1	98.5	99.7	98.6	98.6
Central Kalimantan	5.0	97.5	97.9	97.1	98.2	97.4	97.0
Jakarta	5.0	95.9	96.6	95.3	97.7	96.1	92.6
Banten	5.3	97.7	98.3	97.1	98.3	97.5	97.8
Bangka Belitung	5.4	98.1	98.4	97.7	98.9	97.9	97.7
West Sumatera	6.6	98.0	98.3	97.7	98.5	97.8	98.1
North Kalimantan	7.0	94.6	95.4	93.7	96.4	94.0	95.7
East Kalimantan	7.1	96.2	96.3	96.0	97.2	96.0	95.6
Riau Islands	7.6	94.7	95.3	94.1	96.9	94.2	95.9
Jambi	7.8	98.1	98.2	98.0	98.6	97.9	98.6
North Maluku	7.9	94.5	94.7	94.3	96.2	93.9	96.1
West Java	7.9	98.6	98.8	98.3	99.1	98.4	98.6
West Kalimantan	8.1	94.7	95.0	94.4	95.4	94.4	95.6
North Sulawesi	8.5	97.3	97.7	96.9	98.0	97.3	96.8
Riau	8.8	97.0	97.5	96.4	97.7	96.8	97.0
South Sulawesi	9.8	97.9	97.9	97.9	97.9	97.9	98.0
West Sulawesi	10.3	95.0	95.4	94.6	96.0	94.6	95.7
East Java	10.9	96.0	96.4	95.7	96.8	95.8	96.2
Central Java	10.9	96.9	97.2	96.5	98.1	96.6	96.6
North Sumatera	11.3	95.5	95.9	95.0	96.4	95.2	95.6
Lampung	12.6	92.6	93.2	92.0	95.5	91.7	93.4
Yogyakarta	12.7	94.4	94.4	94.5	96.0	93.9	95.1
Southeast Sulawesi	13.0	96.9	97.3	96.6	97.1	96.8	97.6
South Sumatera	13.1	98.0	98.0	97.9	98.5	97.9	97.8
Central Sulawesi	14.6	95.7	96.1	95.3	96.3	95.4	96.4
West Nusa Tenggara	14.8	96.7	97.7	95.9	96.8	96.7	96.8
Bengkulu	15.0	96.1	96.2	95.9	96.7	95.9	96.5
Aceh	16.4	98.1	98.1	98.0	98.7	97.8	98.6
Gorontalo	16.8	95.7	95.9	95.6	97.0	95.3	96.1
Maluku	21.8	94.2	94.2	94.1	94.8	93.8	95.4
East Nusa Tenggara	22.0	94.9	95.4	94.3	95.9	94.6	94.9
West Papua	26.5	93.1	93.2	92.9	93.6	92.8	94.4
Papua	29.4	96.3	96.5	96.0	96.2	96.3	96.3
Average		96.2	96.5	95.8	97.1	95.9	96.4

Note: FV = fruit and vegetable. Ordered by the average poverty rates (column 1), the provinces in the top box are the richest and those in the bottom box are the poorest. Shaded values show higher than the national average for each group.

**Table 2:** Characteristics of districts and inadequate FV intake among adults in Indonesia, 2018.

	All		Urban		Rural		Difference	
	n	%	n	%	n	%	%	*p*-Value
	(1)	(2)	(3)	(4)	(5)	(6)	(7) = (4–6)	
(a) Characteristics (#)								
Sample size district	514	100%	97	100%	417	100%	0%	
Region								
Papua	95	18.5%	9	9.3%	86	20.6%	11.3%	0.008
Java	128	24.9%	35	36.1%	93	22.3%	−13.8%	
Sumatera	154	30.0%	33	34.0%	121	29.0%	−5.0%	
Kalimantan	56	10.9%	9	9.3%	47	11.3%	2.0%	
Sulawesi	81	15.8%	11	11.3%	70	16.8%	5.4%	
	514		97		417			
Income								
Q1 poor	102	19.8%	3	3.1%	99	23.7%	20.6%	<0.001
Q2	103	20.0%	5	5.2%	98	23.5%	18.3%	
Q3	103	20.0%	13	13.4%	90	21.6%	8.2%	
Q4	103	20.0%	22	22.7%	81	19.4%	−3.3%	
Q5 rich	103	20.0%	54	55.7%	49	11.8%	−43.9%	
	514		97		417			
Education								
Q1 least	103	20.0%	0	0.0%	103	24.7%	24.7%	<0.001
Q2	103	20.0%	11	11.3%	92	22.1%	10.7%	
Q3	103	20.0%	17	17.5%	86	20.6%	3.1%	
Q4	103	20.0%	29	29.9%	74	17.7%	−12.2%	
Q5 most	102	19.8%	40	41.2%	62	14.9%	−26.4%	
	514		97		417			
(b) Inadequate FV intake (%)								
All adults	n/a	96.3%	n/a	95.8%	n/a	96.5%	−0.7%	0.093
Male adults	n/a	96.7%	n/a	96.4%	n/a	96.7%	−0.3%	0.375
Female adults	n/a	96.0%	n/a	95.2%	n/a	96.2%	−1.0%	0.020
Young adults	n/a	97.0%	n/a	97.4%	n/a	97.0%	0.4%	0.309
Adults	n/a	96.1%	n/a	95.4%	n/a	96.3%	−0.9%	0.050
Older adults	n/a	96.7%	n/a	95.2%	n/a	97.0%	−1.8%	<0.001

Note: Q = quintile, n = number, FV = fruit and vegetable, % = proportion of column total, urban = city, rural = regency. Data on district characteristics are from the World Bank and fruit/vegetable intake data are from the Basic Health Survey 2018. For *p*-values, chi-square tests were used in panel (a) and OLS regressions were used in panel (b). See Supplementary Table S4 for the regression outputs.

**Table 3:** Prevalence of inadequate FV intake by region, income, and education among adults in Indonesia, 2018.

	Inadequate FV Intake (N = 514 Districts)					
	All Adults	Males	Females	Young Adults	Adults	Older Adults
Region						
Papua	94.9%	95.1%	94.6%	95.2%	94.6%	95.9%
Sulawesi	96.5%	96.8%	96.3%	97.0%	96.3%	96.9%
Kalimantan	96.8%	97.2%	96.4%	97.6%	96.6%	97.1%
Sumatera	97.2%	97.5%	97.0%	98.0%	97.1%	97.2%
Java	96.1%	96.5%	95.6%	97.0%	95.8%	96.2%
Absolute	1.2%	1.4%	1.0%	1.8%	1.2%	0.3%
Relative	1.01	1.01	1.01	1.02	1.01	1.00
Income						
Q1 poor	95.7%	95.8%	95.6%	95.9%	95.6%	96.5%
Q2	95.9%	96.3%	95.5%	96.6%	95.6%	96.6%
Q3	96.7%	97.1%	96.4%	97.5%	96.5%	96.8%
Q4	96.9%	97.2%	96.5%	97.6%	96.7%	97.0%
Q5 rich	96.5%	96.9%	96.2%	97.6%	96.3%	96.4%
Absolute	0.8%	1.1%	0.6%	1.7%	0.7%	−0.1%
Relative	1.01	1.01	1.01	1.02	1.01	1.00
Education						
Q1 least	96.6%	96.8%	96.5%	96.8%	96.5%	97.2%
Q2	96.5%	96.8%	96.1%	97.1%	96.2%	97.0%
Q3	96.0%	96.3%	95.7%	96.7%	95.8%	96.2%
Q4	96.2%	96.5%	95.9%	97.0%	96.0%	96.5%
Q5 most	96.4%	96.8%	96.0%	97.6%	96.1%	96.4%
Absolute	−0.2%	0.0%	−0.5%	0.8%	−0.4%	−0.8%
Relative	1.00	1.00	0.99	1.01	1.00	0.99

Note: FV = fruit and vegetable, Q = quintile. Java region includes Bali, Papua region includes Maluku and Nusa Tenggara. Income quintile used district-level poverty rate (e.g., Q1 = 20% of districts with highest poverty rate). Absolute (relative) = difference (ratio) between Papua and Java, as well as Q1 and Q5. For education, absolute (relative) was between Q1 and Q5, except among urban (Q2 and Q5). Outcome variables are inadequate FV intake among all adults, males, females, young adults (18–24 years), adults (25–59 years), and older adults (60+ years).

**Table 4:** Multivariate regression results for geographic and socioeconomic disparity in inadequate FV intake among adults in Indonesia, 2018.

	Inadequate FV intake (N = 514 districts)					
	All Adults	Males	Females	Young Adults	Adults	Older Adults
	Coef (*p*-Value)	Coef (*p*-Value)	Coef (*p*-Value)	Coef (*p*-Value)	Coef (*p*-Value)	Coef (*p*-Value)
Region						
Papua	Reference					
Java	1.176 *	1.130 *	1.228 *	1.274 *	1.194 *	0.583
	(0.042)	(0.047)	(0.047)	(0.037)	(0.047)	(0.343)
Sumatera	2.587 **	2.332 **	2.828 **	2.524 **	2.715 **	1.776 **
	(0.000)	(0.000)	(0.000)	(0.000)	(0.000)	(0.003)
Kalimantan	1.691 *	1.617 *	1.714 *	1.633 *	1.740 *	1.365
	(0.020)	(0.023)	(0.027)	(0.032)	(0.021)	(0.077)
Sulawesi	1.859 **	1.647 **	2.075 **	1.618 *	1.956 **	1.262
	(0.002)	(0.006)	(0.001)	(0.011)	(0.002)	(0.051)
Income						
Quintile 1 poor	Reference					
Quintile 2	−0.359	−0.040	−0.683	0.013	−0.491	−0.135
	(0.521)	(0.942)	(0.254)	(0.983)	(0.401)	(0.822)
Quintile 3	0.374	0.653	0.089	0.791	0.297	0.055
	(0.523)	(0.258)	(0.887)	(0.200)	(0.626)	(0.930)
Quintile 4	0.389	0.710	0.058	0.808	0.321	0.094
	(0.507)	(0.218)	(0.926)	(0.191)	(0.598)	(0.881)
Quintile 5 rich	0.341	0.601	0.076	1.014	0.242	−0.301
	(0.585)	(0.328)	(0.909)	(0.123)	(0.710)	(0.651)
Education						
Quintile 1 least	Reference					
Quintile 2	−0.628	−0.482	−0.790	−0.323	−0.750	−0.451
	(0.235)	(0.356)	(0.162)	(0.562)	(0.174)	(0.424)
Quintile 3	−1.171 *	−1.030 *	−1.319 *	−0.763	−1.285 *	−1.336 *
	(0.027)	(0.048)	(0.019)	(0.170)	(0.020)	(0.018)
Quintile 4	−1.059 *	−0.925	−1.195 *	−0.581	−1.197 *	−1.007
	(0.045)	(0.076)	(0.035)	(0.297)	(0.030)	(0.074)
Quintile 5 most	−1.066	−0.855	−1.284 *	−0.262	−1.280 *	−1.246 *
	(0.057)	(0.122)	(0.032)	(0.657)	(0.029)	(0.038)

Note: FV = fruit and vegetable, Coef = coefficient, Q = quintile. Java region includes Bali, Papua region includes Maluku and Nusa Tenggara. Income quintile used district-level poverty rate (e.g., Q1 = 20% of districts with highest poverty rate). Outcome variables are inadequate FV intake among all adults, males, females, young adults (18–24 years), adults (25–59 years), and older adults (60+ years). * = Significant at the 5% level, and ** = significant at the 1% level.

## Data Availability

Available from the authors upon reasonable request.

## Supplementary Materials

The following supporting information can be downloaded at: https://www.mdpi.com/article/10.3390/nu15092160/s1, Figure S1: Map of provinces in Indonesia; Figure S2: Map of Districts by urbanicity, income level, and educational level; Table S1: Urban sample and educational/poverty level by urbanicity and region; Table S2: Ten districts with LOWEST prevalence of inadequate FV intake in Indonesia, 2018; Table S3: Ten districts with HIGHEST prevalence of inadequate FV intake in Indonesia, 2018; Table S4: Regression outputs for urban/rural differences.
