# Supplementary material for: Beyond the Plate: Uncovering Inequalities in Fruit and Vegetable Intake across Indonesian Districts
Source: Nutrients. 2023 Apr 30;15(9):2160. doi: 10.3390/nu15092160 (PMC10180582; doi:10.3390/nu15092160)
Supplement: Supplementary file 1 [file nutrients-15-02160-s001.zip › nutrients-2306528-supplementary.pdf]

Figure S1. Map of provinces in Indonesia

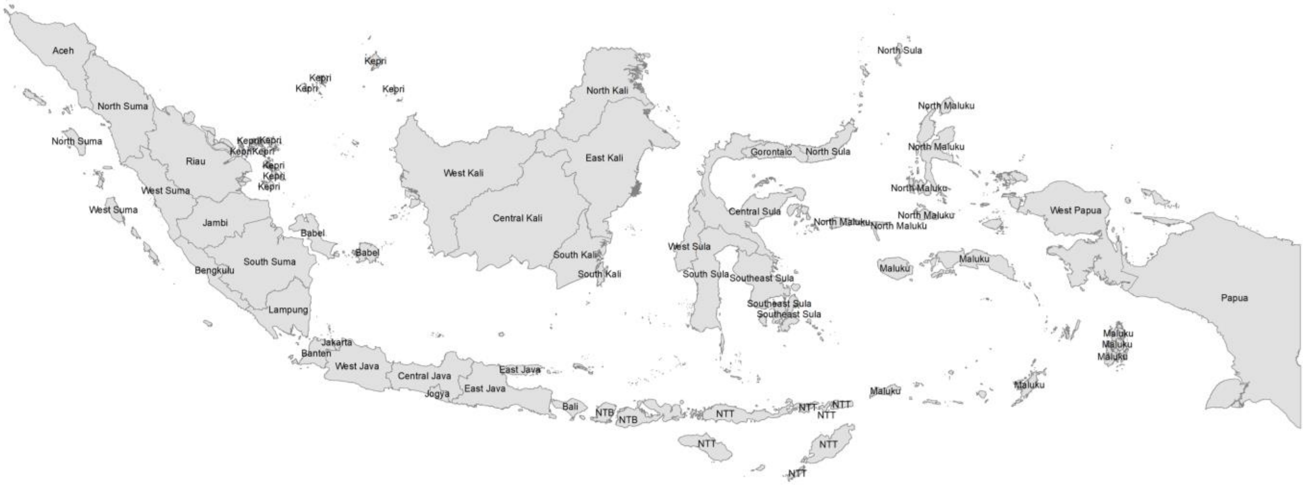

Note: Suma=Sumatera, Kepri=Riau Islands, Sula=Sulawesi, Kali=Kalimantan, NTB=West Nusa Tenggara, NTT=East Nusa Tenggara. We divided the provinces into five regions including Sumatera, Java/Bali, Kalimantan, Sulawesi, and Papua/Maluku/Nusa Tenggara. Java/Bali is the most developed and Papua/Maluku/Nusa Tenggara is the least developed. We obtained the shapefile from the Indonesian Information and Geospatial Agency and created the map in ArcMap 10.

Figure S2. Map of Districts by urbanicity, income level, and educational level

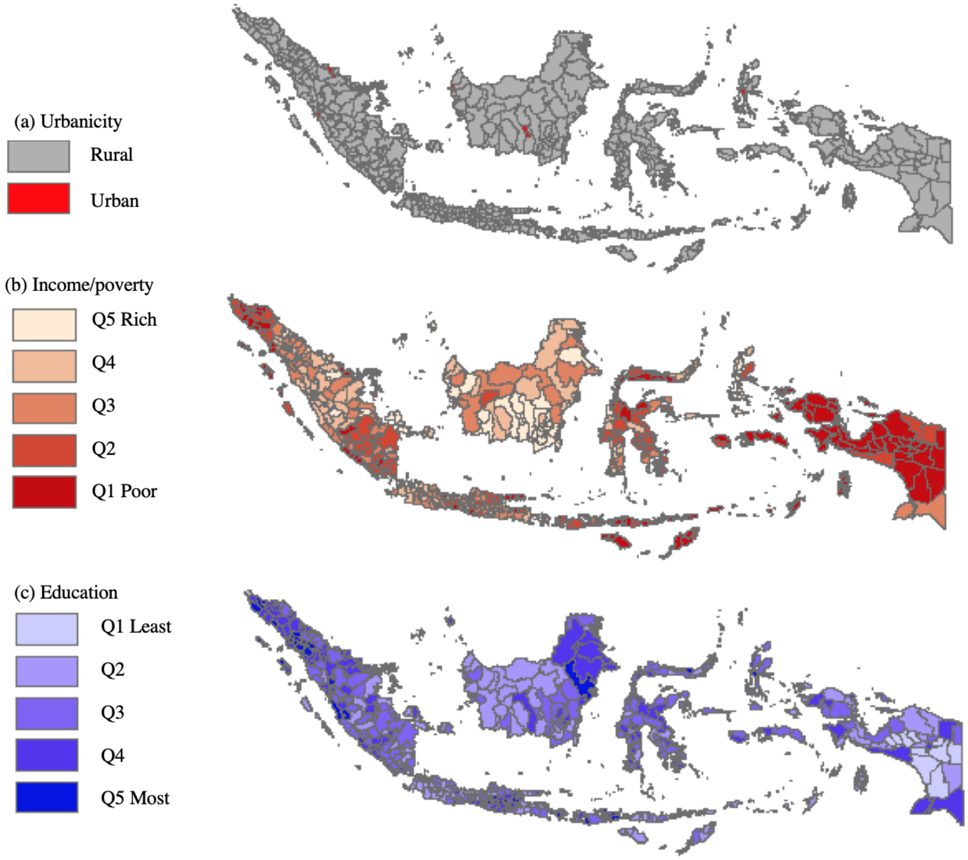

Table S1. Urban sample and educational/poverty level by urbanicity and region

(a)

Urbanicity	Sample N (%)	Education enrolment ratio (%)	Poverty rates (%)
Urban	97 (18.9)	68.5	6.9
Rural	417 (81.1)	59.6	13.6
Total	514 (100)	61.3	12.3

(b)

Region	Urban (%)	Education enrolment ratio (%)	Poverty rates (%)
Papua	9.5	53.8	22.6
Sulawesi	13.6	61.4	11.6
Kalimantan	16.1	57.2	6.3
Sumatera	21.4	66.2	11.2
Java	27.3	62.6	9.3
Total	61.3	61.3	12.3

Table S2. Ten districts with LOWEST prevalence of inadequate FV intake in Indonesia, 2018

	Prevalence	Province	Region	Urban	Poverty	Education	Pop (000)
(a) All adults							
Kab. Kolaka Timur	70.1%	Southeast Sulawesi	Sulawesi	Rural	14%	64%	178
Nagekeo	75.4%	East Nusa Tenggara	Papua	Rural	13%	51%	139
Kab. Tambrauw	76.8%	West Papua	Papua	Rural	35%	47%	14
Kab. Tuban	77.9%	East Java	Java	Rural	15%	56%	1152
Kab. Sorong	81.1%	West Papua	Papua	Rural	30%	66%	80
Kota Sibolga	82.7%	North Sumatera	Sumatera	Urban	12%	74%	86
Kab. Ngada	82.8%	East Nusa Tenggara	Papua	Rural	13%	61%	155
Kota Pontianak	83.5%	West Kalimantan	Kalimantan	Urban	5%	61%	607
Kab. Lembata	84.5%	East Nusa Tenggara	Papua	Rural	26%	51%	132
Kab. Mambramo Tengah	85.1%	Papua	Papua	Rural	37%	54%	46
AVERAGE					**20%**	**59%**	**259**
(b) Male adults							
Kab. Kolaka Timur	71%	Southeast Sulawesi	Sulawesi	Rural	14%	64%	178
Kab. Tambrauw	71%	West Papua	Papua	Rural	35%	47%	14
Kab. Sorong	77%	West Papua	Papua	Rural	30%	66%	80
Nagekeo	78%	East Nusa Tenggara	Papua	Rural	13%	51%	139
Kab. Tuban	80%	East Java	Java	Rural	15%	56%	1152
Kab. Mambramo Tengah	83%	Papua	Papua	Rural	37%	54%	46
Kota Pontianak	84%	West Kalimantan	Kalimantan	Urban	5%	61%	607
Kota Sibolga	84.5%	North Sumatera	Sumatera	Urban	12%	74%	86
Kab. Lembata	84.8%	East Nusa Tenggara	Papua	Rural	26%	51%	132
Kab. Ngada	85.2%	East Nusa Tenggara	Papua	Rural	13%	61%	155
AVERAGE					**20%**	**59%**	**259**
(c) Female adults							
Kab. Kolaka Timur	69%	Southeast Sulawesi	Sulawesi	Rural	14%	64%	178
Nagekeo	73%	East Nusa Tenggara	Papua	Rural	13%	51%	139
Kab. Tuban	76%	East Java	Java	Rural	15%	56%	1152
Kab. Ngada	81%	East Nusa Tenggara	Papua	Rural	13%	61%	155
Kota Sibolga	80.9%	North Sumatera	Sumatera	Urban	12%	74%	86
Kab. Tambrauw	82.8%	West Papua	Papua	Rural	35%	47%	14
Kota Pontianak	83.0%	West Kalimantan	Kalimantan	Urban	5%	61%	607
Kab. Lembata	84.3%	East Nusa Tenggara	Papua	Rural	26%	51%	132
Kab. Konawe Utara	84.9%	Southeast Sulawesi	Sulawesi	Rural	14%	54%	58
Kab. Buru	85.2%	Maluku	Papua	Rural	17%	62%	128
AVERAGE					**16%**	**58%**	**265**
(d) Young adults							
Kab. Kolaka Timur	67%	Southeast Sulawesi	Sulawesi	Rural	14%	64%	178
Kab. Tambrauw	72%	West Papua	Papua	Rural	35%	47%	14
Nagekeo	77%	East Nusa Tenggara	Papua	Rural	13%	51%	139
Kab. Sorong	78%	West Papua	Papua	Rural	30%	66%	80
Kab. Ngada	80%	East Nusa Tenggara	Papua	Rural	13%	61%	155
Kab. Maybrat	80%	West Papua	Papua	Rural	33%	69%	37
Kab. Tuban	81%	East Java	Java	Rural	15%	56%	1152
Kab. Konawe Utara	84%	Southeast Sulawesi	Sulawesi	Rural	14%	54%	58
Kota Pontianak	84%	West Kalimantan	Kalimantan	Urban	5%	61%	607
Kab. Kaimana	85%	West Papua	Papua	Rural	17%	52%	54
AVERAGE					**19%**	**58%**	**247**
(e) Adults							
Kab. Kolaka Timur	69.1%	Southeast Sulawesi	Sulawesi	Rural	14%	64%	178
Kab. Tuban	75.1%	East Java	Java	Rural	15%	56%	1152
Nagekeo	75.6%	East Nusa Tenggara	Papua	Rural	13%	51%	139
Kab. Tambrauw	77.8%	West Papua	Papua	Rural	35%	47%	14
Kab. Sorong	81.3%	West Papua	Papua	Rural	30%	66%	80
Kota Sibolga	82.7%	North Sumatera	Sumatera	Urban	12%	74%	86
Kab. Ngada	82.9%	East Nusa Tenggara	Papua	Rural	13%	61%	155
Kota Pontianak	83.0%	West Kalimantan	Kalimantan	Urban	5%	61%	607

Kab. Lembata	83.0%	East Nusa Tenggara	Papua	Rural	26%	51%	132
Kab. Mambramo Tengah	84.4%	Papua	Papua	Rural	37%	54%	46
AVERAGE					**20%**	**59%**	**259**
(f) Older adults							
Kota Sibolga	72.2%	North Sumatera	Sumatera	Urban	12%	74%	86
Nagekeo	73.8%	East Nusa Tenggara	Papua	Rural	13%	51%	139
Kab. Buru	74.9%	Maluku	Papua	Rural	17%	62%	128
Kab. Tambrauw	78.1%	West Papua	Papua	Rural	35%	47%	14
Kab. Teluk Bintuni	80.0%	West Papua	Papua	Rural	31%	56%	59
Kab. Keerom	80.1%	Papua	Papua	Rural	17%	61%	54
Kab. Kolaka Timur	81.2%	Southeast Sulawesi	Sulawesi	Rural	14%	64%	178
Kota Padang Panjang	84.5%	West Sumatera	Sumatera	Urban	6%	74%	51
Kab. Sorong	84.6%	West Papua	Papua	Rural	30%	66%	80
Kota Mataram	84.7%	West Nusa Tenggara	Papua	Urban	9%	74%	449
AVERAGE					**18%**	**63%**	**124**

Note: FV=fruit & vegetable, Urban=City, Rural=Regency; Pop=Population. The districts are ordered by prevalence (column 1).

Table S3. Ten districts with HIGHEST prevalence of inadequate FV intake in Indonesia, 2018

	Prevalence	Province	Region	Urban	Poverty	Education	Pop (000)
(a) All adults							
Kab. Mamasa	99.9%	West Sulawesi	Sulawesi	Rural	13%	66%	152
Kab. Pangkep	99.9%	South Sulawesi	Sulawesi	Rural	15%	59%	323
Kab. Simalungun	100.0%	North Sumatera	Sumatera	Rural	9%	63%	849
Kab. Nias Utara	100.0%	North Sumatera	Sumatera	Rural	27%	73%	134
Kab. Konawe Kepulauan	100.0%	Southeast Sulawesi	Sulawesi	Rural	17%	63%	32
Kab. Buton Selatan	100.0%	Southeast Sulawesi	Sulawesi	Rural	15%	44%	77
Kab. Yahukimo	100.0%	Papua	Papua	Rural	39%	12%	181
Kab. Tolikara	100.0%	Papua	Papua	Rural	33%	34%	131
Kab. Dogiyai	100.0%	Papua	Papua	Rural	30%	39%	92
Kab. Diyai	100.0%	Papua	Papua	Rural	43%	51%	69
AVERAGE					**24%**	**51%**	**204**
(b) Male adults							
Kab. Simalungun	100.0%	North Sumatera	Sumatera	Rural	9%	63%	849
Kab. Nias Utara	100.0%	North Sumatera	Sumatera	Rural	27%	73%	134
Kab. Kerinci	100.0%	Jambi	Sumatera	Rural	7%	77%	235
Kab. Batang Hari	100.0%	Jambi	Sumatera	Rural	10%	66%	260
Kab. Tanah Tidung	100.0%	North Kalimantan	Kalimantan	Rural	5%	45%	22
Kab. Konawe Kepulauan	100.0%	Southeast Sulawesi	Sulawesi	Rural	17%	63%	32
Kab. Buton Selatan	100.0%	Southeast Sulawesi	Sulawesi	Rural	15%	44%	77
Kab. Sorong Selatan	100.0%	West Papua	Papua	Rural	19%	56%	43
Kab. Yahukimo	100.0%	Papua	Papua	Rural	39%	12%	181
Kab. Tolikara	100.0%	Papua	Papua	Rural	33%	34%	131
Kab. Mambramo Raya	100.0%	Papua	Papua	Rural	30%	51%	21
Kab. Puncak	100.0%	Papua	Papua	Rural	38%	9%	103
Kab. Dogiyai	100.0%	Papua	Papua	Rural	30%	39%	92
Kab. Diyai	100.0%	Papua	Papua	Rural	43%	51%	69
AVERAGE					**23%**	**49%**	**161**
(c) Female adults							
Kab. Simalungun	100.0%	North Sumatera	Sumatera	Rural	9%	63%	849
Kab. Padang Lawas Utara	100.0%	North Sumatera	Sumatera	Rural	10%	69%	252
Kab. Nias Utara	100.0%	North Sumatera	Sumatera	Rural	27%	73%	134
Kab. Balangan	100.0%	South Kalimantan	Kalimantan	Rural	6%	65%	123
Kab. Konawe Kepulauan	100.0%	Southeast Sulawesi	Sulawesi	Rural	17%	63%	32
Kab. Buton Selatan	100.0%	Southeast Sulawesi	Sulawesi	Rural	15%	44%	77
Kab. Mamasa	100.0%	West Sulawesi	Sulawesi	Rural	13%	66%	152
Kab. Asmat	100.0%	Papua	Papua	Rural	27%	21%	88
Kab. Yahukimo	100.0%	Papua	Papua	Rural	39%	12%	181
Kab. Tolikara	100.0%	Papua	Papua	Rural	33%	34%	131
Kab. Dogiyai	100.0%	Papua	Papua	Rural	30%	39%	92
Kab. Diyai	100.0%	Papua	Papua	Rural	43%	51%	69
AVERAGE					**23%**	**50%**	**182**
(d) Young adults							
Kab. Jayawijaya	100.0%	Papua	Papua	Rural	39%	67%	206
Kab. Paniayi	100.0%	Papua	Papua	Rural	37%	25%	164
Kab. Asmat	100.0%	Papua	Papua	Rural	27%	21%	88
Kab. Yahukimo	100.0%	Papua	Papua	Rural	39%	12%	181
Kab. Tolikara	100.0%	Papua	Papua	Rural	33%	34%	131
Kab. Waropen	100.0%	Papua	Papua	Rural	31%	61%	28
Kab. Mambramo Raya	100.0%	Papua	Papua	Rural	30%	51%	21
Kab. Puncak	100.0%	Papua	Papua	Rural	38%	9%	103
Kab. Dogiyai	100.0%	Papua	Papua	Rural	30%	39%	92
Kab. Diyai	100.0%	Papua	Papua	Rural	43%	51%	69
AVERAGE					**35%**	**37%**	**108**
(e) Adults							
Kab. Mamasa	99.9%	West Sulawesi	Sulawesi	Rural	13%	66%	152

Kab. Pangkep	100.0%	South Sulawesi	Sulawesi	Rural	15%	59%	323
Kab. Simalungun	100.0%	North Sumatera	Sumatera	Rural	9%	63%	849
Kab. Nias Utara	100.0%	North Sumatera	Sumatera	Rural	27%	73%	134
Kab. Konawe Kepulauan	100.0%	Southeast Sulawesi	Sulawesi	Rural	17%	63%	32
Kab. Buton Selatan	100.0%	Southeast Sulawesi	Sulawesi	Rural	15%	44%	77
Kab. Yahukimo	100.0%	Papua	Papua	Rural	39%	12%	181
Kab. Tolikara	100.0%	Papua	Papua	Rural	33%	34%	131
Kab. Dogiyai	100.0%	Papua	Papua	Rural	30%	39%	92
Kab. Diyai	100.0%	Papua	Papua	Rural	43%	51%	69
AVERAGE					**24%**	**51%**	**204**
(f) Older adults							
Kab. Mambramo Raya	100.0%	Papua	Papua	Rural	30%	51%	21
Kab. Sorong Selatan	100.0%	West Papua	Papua	Rural	19%	56%	43
Kab. Manowari Selatan	100.0%	West Papua	Papua	Rural	31%	47%	22
Kab. Kerinci	100.0%	Jambi	Sumatera	Rural	7%	77%	235
Kab. Buton Selatan	100.0%	Southeast Sulawesi	Sulawesi	Rural	15%	44%	77
Kab. Puncak Jaya	100.0%	Papua	Papua	Rural	36%	21%	115
Kab. Sintang	100.0%	West Kalimantan	Kalimantan	Rural	10%	45%	396
Kab. Anambas Kep	100.0%	Riau Islands	Sumatera	Rural	7%	77%	40
Kab. Sijunjung	100.0%	West Sumatera	Sumatera	Rural	7%	59%	222
Kab. Halmahera Tengah	100.0%	North Maluku	Papua	Rural	14%	63%	50
Kab. Boven Digul	100.0%	Papua	Papua	Rural	20%	35%	63
Kab. Padang Pariaman	100.0%	West Sumatera	Sumatera	Rural	8%	72%	406
Sabu Raijua	100.0%	East Nusa Tenggara	Papua	Rural	31%	69%	86
Kab Aceh Tamiang	100.0%	Aceh	Sumatera	Rural	14%	66%	278
kab. Tanah Bumbu	100.0%	South Kalimantan	Kalimantan	Rural	5%	62%	324
AVERAGE					**17%**	**56%**	**159**

Note: FV=fruit & vegetable, Urban=City, Rural=Regency; Pop=Population. The districts are ordered by prevalence (column 1).

Table S4. Regression outputs for urban/rural differences

	Inadequate FV intake					
	All adults Coef	Males Coef	Females Coef	18-24 yrs Coef	25-59 yrs Coef	60+ yrs Coef
Rural	Reference					
Urban	-0.70	-0.36	-1.04*	0.45	-0.85	-1.81**
Constant	96.48**	96.72**	96.24**	96.95**	96.29**	96.99**
Observations	514	514	514	514	514	514
R-squared	0.01	0.00	0.01	0.00	0.01	0.03

Note: FV=fruit & vegetable, Coef = OLS Coefficient; Significance level ** p<0.01, * p<0.05
